# An Impedance-Based Immunosensor for the Detection of Ovalbumin in White Wine

**DOI:** 10.3390/bios13070669

**Published:** 2023-06-22

**Authors:** Alessia Calabrese, Alessandro Capo, Angela Capaccio, Elettra Agovino, Antonio Varriale, Michelangelo Pascale, Sabato D’Auria, Maria Staiano

**Affiliations:** 1 Institute of Food Science, URT-CNR, 80126 Naples, Italy; alessia.calabrese@isa.cnr.it (A.C.); alessandro.capo@isa.cnr.it (A.C.); angela.capaccio@isa.cnr.it (A.C.); e.agovino95@libero.it (E.A.); antonio.varriale@isa.cnr.it (A.V.); 2 Institute of Food Science, CNR, 83100 Avellino, Italy; michelangelo.pascale@cnr.it (M.P.); maria.staiano@isa.cnr.it (M.S.); 3 Department of Biology, Agriculture and Food Science, CNR, Piazzale Aldo Moro, 7, 00185 Rome, Italy

**Keywords:** allergen, wine, ovalbumin (OVA), molecular recognition element (MRE), electrochemical impedance spectroscopy (EIS), food safety, immunosensor, biosensors

## Abstract

Food allergies are an exceptional response of the immune system caused by the ingestion of specific foods. The main foods responsible for allergic reactions are milk, eggs, seafood, soy, peanuts, tree nuts, wheat, and their derived products. Chicken egg ovalbumin (OVA), a common allergen molecule, is often used for the clarification process of wine. Traces of OVA remain in the wine during the fining process, and they can cause significant allergic reactions in sensitive consumers. Consequently, the European Food Safety Authority (EFSA) and the American Food and Drug Administration (FDA) have shown the risks for allergic people to assume allergenic foods and food ingredients, including eggs. Commonly, OVA detection requires sophisticated and time-consuming analytical techniques. Intending to develop a faster assay, we designed a proof-of-concept non-Faradaic impedimetric immunosensor for monitoring the presence of OVA in wine. Polyclonal antibodies anti-OVA were covalently immobilised onto an 11-mercaptoundecanoic-acid (11-MUA)-modified gold surface. The developed immunosensor was able to detect OVA in diluted white wine without the need for an external probe or any pre-treatment step with a sensitivity of 0.20 µg/mL, complying with the limit established by the resolution OIV/COMEX 502–2012 for the quantification of allergens in wine.

## 1. Introduction

Food allergies are an abnormal body immunological reaction to the ingestion of specific foods. Milk of cows, eggs, shellfish, soy, peanuts, tree nuts, and wheat are the most-common allergenic foods. The most-efficient approach to prevent allergy is to avoid the dietary intake of such foods. However, the ubiquity of derivate food components in cooked or manufactured food products makes it challenging to avoid allergy reactions. Moreover, the body exposure to these food components may lead to a different level of allergy, from low symptoms (rush, asthma) to severe conditions such as anaphylactic responses. In this context, egg-derived products containing allergic proteins are widely employed in winemaker processing. Chicken egg ovalbumin (OVA) is one of the main proteins used in winemaking in the fining process [1]. This procedure allows for the clearing and the biochemical stabilisation of wine through the precipitation of tiny floating particles, bacteria, tartrates, proteins, pectins, various tannins, and other phenolic compounds [2]. The fining proteins can be removed by decantation, filtration steps, or a secondary fining procedure using inorganic agents, such as bentonite [3]. This operation should lead to a non-allergenic product, even if the risk for wine consumers that are allergic to egg proteins cannot be entirely excluded. An opinion paper by the European Food Safety Authority (EFSA) has highlighted the potential risks for allergic people to assume traces of OVA remained in wine during the fining process, causing significant allergic reactions [4]. Moreover, the Food and Drug Administration (FDA) in the USA has regulated the utilisation of egg products to protect consumers from the possible consequences of accidental egg consumption [5].

Within the European Union, wine producers must specify in the ingredient label the presence of allergenic aids from animal origin added for the fining process, as regulated by Directive 2007/68/EC [6]. As a result, developing accurate and sensitive approaches for detecting egg proteins in food matrices represents an opportunity for producers to identify the risk associated with residual allergens. To offset the lack of reference analytical methods for detecting fining agent proteins, the International Organization of Vine and Wine (OIV) established the analytical requirements for methods under development [7]. More precisely, the enzyme-linked immunosorbent assay (ELISA) must fulfil a detection limit of ≤0.25 µg/mL. This technique is the most-routinely used for monitoring proteins as allergenic compounds in food samples. However, besides requiring experienced personnel, in some cases, the ELISA test needs more sensitivity for allergen detection in some food matrices [8]. On the contrary, liquid chromatography coupled with mass spectrometry (LC-MS and LC-MS/MS) allows for complete and simultaneous identification of the analytes, representing a robust technique for detecting residual fining agents [9,10,11,12]. Nonetheless, this technique relies on time-consuming sample preparation, complicated data analysis, and costly instrumentation. In this context, biosensors might help to assess the residual presence of allergens allowing in situ and real-time food safety monitoring. A recent review reported the most-relevant progress in biosensor development for food-allergen management [13]. To date, several biosensors to detect food allergens have been proposed [14,15,16,17,18,19]. They show LoD values ranging from pg/mL to μg/mL and only a few of them have been tested on wines [18,19].

Electrochemical-impedance-based biosensors present various advantages, such as the affordability and the simplicity of miniaturisation. In particular, non-Faradaic-based biosensors, also known as capacitive sensors, detect the capacitance variations at the electrode surface produced by the molecular binding events. These sensors are well-suited to point-of-care applications thanks to their potential, such as rapid response, portability, and ease of use. Furthermore, unlike Faradaic biosensors, which require adding a potentially harmful redox probe, which could damage biomolecules [20], the non-Faradaic approach is label-free, allowing the analysis without sample preparation or trained personnel [21,22].

In this work, we explored the application of electrochemical impedance spectroscopy (EIS) to develop a non-Faradaic impedimetric immunosensor for detecting the presence of OVA in white wine. For this purpose, polyclonal antibodies against OVA (pAb anti-OVA) were produced and characterised by an indirect ELISA test. Then, the antibodies were covalently attached to a gold-based electrode surface. Atomic force microscopy (AFM) was employed to study the surface topography of the functionalised biosensor surfaces. Finally, EIS was used to characterise the performance of the immunosensor, which allowed for a simple and fast determination of OVA directly in diluted white wine without any additional reagent.

## 2. Materials and Methods

### 2.1. Reagents and Instruments

All commercially available reagents were chosen for the highest quality. 11-mercaptoundecanoic acid (11-MUA), N-hydroxysuccinimide (NHS), N-(3-dimethylaminopropyl)-N’-ethylcarbodiimide hydrochloride (EDC), sulfuric acid (H_2_SO_4_), ethanolamine (ETA), ethanol, albumin from chicken egg (OVA), and 3,5-tetramethylbenzidine (TMB) were purchased from Sigma-Aldrich (Milan, Italy). Goat polyclonal to rabbit IgG-HRP conjugate (secondary antibody) was from Abcam (Cambridge, U.K.). The antibodies against OVA were produced and purchased from Covalab SAS (Bron, France). nProtein A Sepharose 4 Fast Flow resin for antibody purification was acquired from Cytiva (Washington, DC, USA). Materials used for protein electrophoresis were obtained from Bio-Rad (Hercules, CA, USA). Microplates (96-well), Nunc LockWell MaxiSorp form, and a microplate reader, Tecan Infinity 200 Pro (Tecan, Männedorf, Switzerland), were used for the indirect ELISA. UV measurements were carried out on a Jasco V-730 UV/Vis spectrophotometer. Ultrafiltration units Vivaspin^®^ Turbo 15 (30,000 MWCO) for antibody concentration were from Sartorius (Göttingen, Germany). The miniaturised All-in-One electrochemical workstation (MicruX ECStat), the All-in-One cell, the batch-cell Add-on, and thin-film gold single electrodes (ED-SE1-Au) were purchased from Micrux Technologies (Oviedo, Spain). White wine (alcohol content: 10.5%) was acquired from a local market.

### 2.2. Antibody Production and Purification

Antibodies anti-OVA were produced by Covalab (France), according to Varriale et al. (2016) [23]. In brief, two rabbits were immunised following a standard protocol by intradermal inoculation of an antigen (0.5 mg of OVA per rabbit). After the immunisation period, the rabbits were sacrificed. Their blood was recovered and centrifuged to separate the blood cells from the serum. From the obtained serum, the antibodies were purified, according to Pennacchio et al. (2016) [24]. In particular, 1 mL of rabbit serum diluted with 1.0 mL of sodium phosphate (NaP) 20 mM, pH 7.0, was applied to a Protein A column. Then, the IgG fraction was eluted with sodium citrate 0.1 M, pH 3.0, and immediately buffered in sodium borate 1 M, pH 8.5. Finally, the IgG concentration and purity were checked by absorbance measurement at 278 nm and SDS-PAGE (12% acrylamide), respectively. The obtained pure samples were concentrated at 1.6 mg/mL by centrifugal concentrators.

### 2.3. Indirect ELISA

In order to verify the binding capacity of the produced antibodies, an indirect ELISA was performed, according to Capo et al. (2022) [25]. The antigen OVA was dissolved in carbonate buffer (coating buffer) 0.05 M, pH 9.6, and diluted from 0.005 µg/mL to 50 µg/mL. The 50 μL/well of each dilution was used to coat 96-well microplates, incubated overnight at 4 °C. Coating buffer and bovine serum albumin (BSA) (100 μg/mL) were used as controls. The plate was rinsed thrice with TBS-T (TBS 0.01 M, pH 7.4; 0.05% Tween-20), incubated with 200 μL/well of blocking buffer (TBS; 5% *w*/*v* non-fat dried milk) at 37 °C for 2 h, and rewashed three times with TBS-T. Afterward, pAb anti-OVA 1 μg/mL (50 μL/well) diluted in blocking buffer (TBS; 1% non-fat dried milk; 0.05% *v*/*v* Tween-20; pH 7.4) was incubated at 37 °C for 2 h. After three steps of washing with TBS-T, 50 μL/well of horseradish-peroxidase (HRP)-conjugated goat anti-rabbit IgG antibodies (0.5 μg/mL) was diluted in blocking buffer (TBS; 1% non-fat dried milk; 0.05% *v*/*v* Tween-20; pH 7.4) and incubated for 1 h at 37 °C. After an incubation of 10 min at 37 °C with the TMB substrate (100 μL/well), the stopping solution (HCl 2.5 M; 50 μL/well) was added to stop the colour development, and the absorbance was recorded at 450 nm.

### 2.4. Immunosensor Development

**Figure 1:**
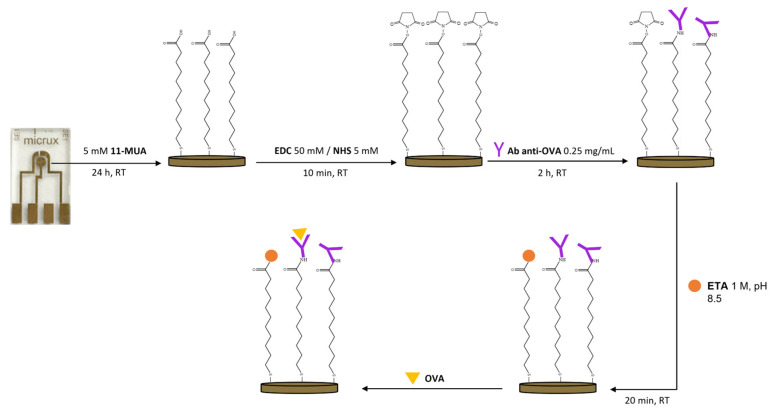
Schematic diagram of the immunosensor fabrication. Gold thin-film electrochemical sensors (ED-SE1-Au) were treated sequentially with 11-MUA, a mixture of NHS and EDC, a solution of pAb anti-OVA, and ETA. The immunosensor was tested at different concentrations of OVA.

The electrochemical sensors used in this work were gold-based and consisted of a three-electrode configuration (reference, working, and counter, as shown in Figure 1 from the left to the right of the chip). Before the derivatisation procedure, an electrochemical surface pre-cleaning was performed through 12 potential cycles in the range of −1.0 to +1.3 V, at a scan rate of 0.1 V/s, in the presence of sulfuric acid (H_2_SO_4_) (0.05 M (5 μL)). Next, the clean gold substrates were immersed in an ethanolic solution of 11-MUA 5 mM (350 μL) for 24 h [26]. Afterward, the antibodies were immobilised on the working electrode via carbodiimide-mediated coupling in two consecutive steps: (1) a 10 min incubation with a mixture of EDC/NHS (5 μL) (50 mM/5 mM) (volume ratio 1:1) in phosphate-buffered saline (PBS, pH 7.4); (2) a 2 h incubation with pAb anti-OVA solution (0.25 mg/mL (5 μL)). Lastly, the unreacted active sites were blocked with ethanolamine 1 M (pH 8.5, 5 μL) for 20 min (Figure 1).

### 2.5. Surface Characterisation by Atomic Force Microscopy

The immobilisation of the pAb anti-OVA on the gold impedimetric surface was characterised by AFM. The AFM images were acquired using a Ntegra Prima system (NT-MDT Spectrum Instruments, Zelenograd Russia) equipped with an AFM silicon tip (NSG01, NT-MDT) having a spring constant of 5 N/m and a nominal resonance frequency of 160 KHz in air. AFM measurements were carried out in tapping mode and in the PBS buffer to preserve the structure and function of the biological components. For each sample, random 1 μm × 1 μm maps were scanned throughout the working area of the gold electrode with a resolution of 200 pixels per line and a scan rate of 2 Hz. From the topographic images, the root-mean-squared roughness (RMS) was calculated and averaged on ten AFM scans after image processing with the open-source software Gwyddion 2.62.

### 2.6. Impedimetric Measurements

Non-Faradaic impedance spectroscopy is able to investigate antigen–antibody biorecognition events without the use of an external redox probe.

The measuring cell used consisted of an All-in-One platform, which enabled the use of the thin-film electrodes in static conditions, with a batch-cell Add-on, which provided an interface with the electrochemical workstation, and it facilitated the dropping of the sample on the electrode.

For this purpose, the working area of the immunosensor was incubated for 10 min with 5 μL of OVA dissolved in PBS (pH 7.4) at different concentrations (0.001, 0.005, 0.01, 0.05, 0.1, and 0.5 μg/mL).

After thoroughly washing the surfaces with PBS, impedimetric measurements were performed in PBS at 25 °C, using a sinusoidal AC potential (0.1 V) and a DC potential of 0 V in the frequency range of 0.1 to 100,000 Hz. The AC potential value was chosen after analysing the impedance response of the system at different values (0.005, 0.01, 0.05, and 0.1 V) (Figure S1). To test the cross-reactivity of the immunosensor, BSA at increasing concentrations was used as a negative control (Figure S2).

### 2.7. Preparation of Wine Samples for Impedimetric Tests on Real Matrix

In order to test the performance of the immunosensor on a real matrix, a white wine acquired in a local grocery was diluted 1:200 in PBS (pH 7.4) and was spiked with different concentrations of OVA (0.001, 0.005, 0.01, 0.05, 0.1, and 0.5 μg/mL). The dilution ratio was chosen to minimise matrix effects, preserving the characteristics of the immunosensor [18]. The working area of the electrode was incubated for 10 min with 5 μL of the sample. After extensive washing steps, impedimetric measurements were performed as previously described.

### 2.8. Statistical Analysis

All the measurements were carried out in triplicate.

The standard deviation (SD) for all the data reported was calculated from the formula:

(1) $$\mathrm{SD}=\sqrt{\frac{{\Sigma (x-\bar{x})}^{2}}{\left(n-1\right)}},$$

where *x* is the sample mean average and *n* is the sample size.

The limit of detection (LoD) for indirect ELISA was calculated according to Ambruster et al. (2008) (LoD = LoB + 1.645 (SD low concentration OVA) [27] and for the immunosensor performance following Shrivastava (2011) (LoD = 3.3 S/b) [28]. Data were analysed in Microsoft^®^ Excel 2016 and Origin^®^ 2018.

## 3. Results and Discussion

In this work, pAb anti-OVA were used to develop a non-Faradaic impedimetric immunosensor to detect the presence of traces of ovalbumin in white wine.

### 3.1. Evaluation of the Antibody-Binding Capacity by Indirect ELISA

**Figure 2:**
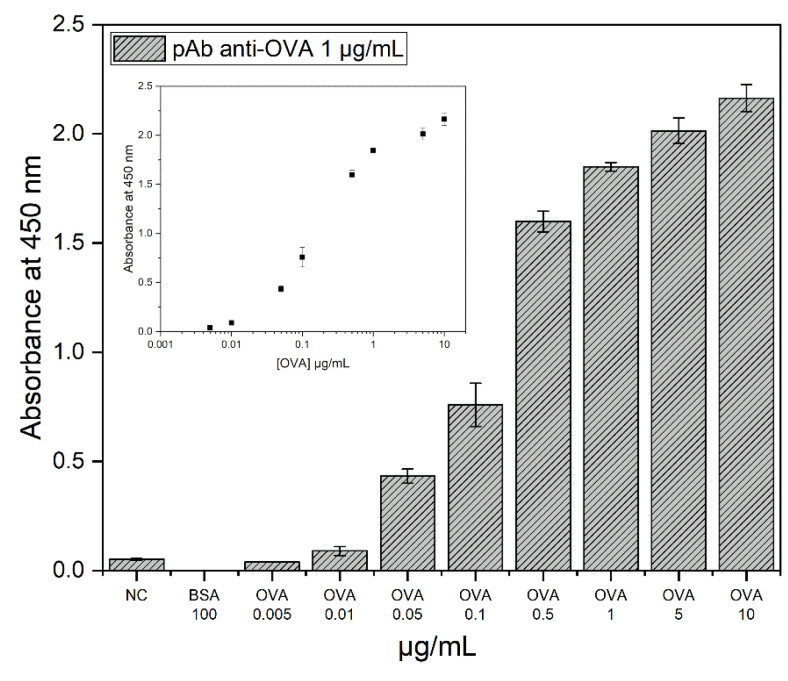
Indirect ELISA results for pAb anti-OVA (1 μg/mL) binding capacity. The figure shows that OVA was recognised by the pAbs anti-OVA up to 0.05 µg/mL (NC: no coating; BSA used as a negative control). The insert shows the variation of signal at 450 nm as function of the ovalbumin concentration.

After the Ab purification procedure (described in Section 2.2), the binding capacity of the pAb anti-OVA was assessed by an indirect ELISA test. Figure 2 shows that the pAbs anti-OVA were able to recognise the antigen up to 0.05 µg/mL. The limit of detection (LoD), determined by considering the measured limit of blank (LoB) and the replicates (*n* = 3) of the sample containing a low concentration of analyte LoD = LoB + 1.645 (SD low-concentration OVA) [27], was estimated to be 0.09 μg/mL.

### 3.2. Electrochemical Characterisation of the Immunosensor Assembling

A typical biorecognition surface for capacitance detection comprises two layers: a double insulation layer and a recognition layer; a third layer is generated by analyte binding to the recognition element. A high-capacitance biorecognition layer detects slight variations induced by the binding event [29,30]. Thus, achieving a tightly packed biorecognition layer (SAM: self-assembled monolayer of long thiols C11–C16) allows for maximising the ability to detect small impedance and capacity variations, reducing unrestricted ionic migration at the interface.

The pAbs anti-OVA were covalently immobilised on an 11-MUA-modified gold surface SAM (Section 2.4). The electrode derivatisation for the immunosensor assembling was monitored using non-Faradaic electrochemical impedance spectroscopy (EIS), characterised by resistive and capacitive contributions. A non-Faradaic system directly detects the analyte without needing a redox probe, such as a ferrocyanide–ferricyanide redox couple (Fe (CN)_6_^3−/4−^). As a result, the experimental process turns out to be more manageable and suitable for rapid monitoring.

**Figure 3:**
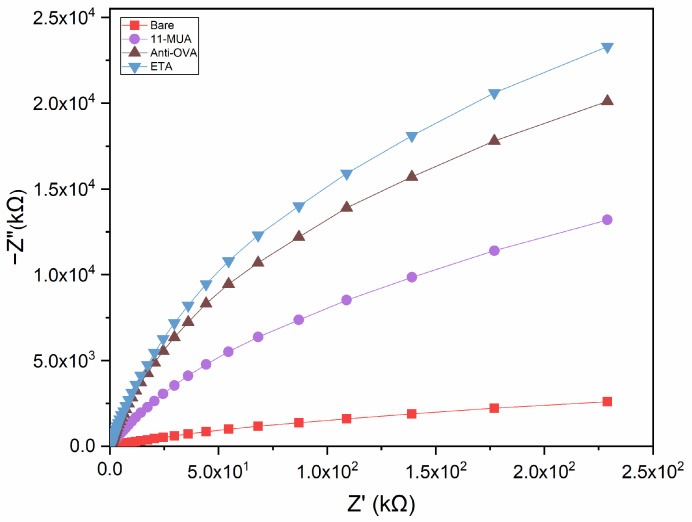
Nyquist plots for the different functionalisation steps performed for the fabrication of the impedimetric immunosensor. The impedance of the electrochemical system increased after each step of the functionalisation procedure.

The impedance data are presented in the Nyquist plot, where the imaginary part (Z″) is plotted versus the real part (Z′) of an impedance Z over a specified frequency range (0.1 to 100,000 Hz). Due to the absence of a redox probe, a non-Faradaic Nyquist plot shows a large incomplete semicircle, lacking the parameters related to electron transfer, including charge transfer resistance (R_ct_) and Warburg impedance (Z_W_). Therefore, the impedance of a non-Faradaic sensor is determined by the insulating characteristics of the species bond to the conductive substrate. As a result, the deposition of the consecutive layers on the electrode causes an increase in the impedance of the system (Figure 3). This variation could be attributed to the coating layer on the electrode surface, which increases during the assembly steps.

### 3.3. AFM Analysis

**Figure 4:**
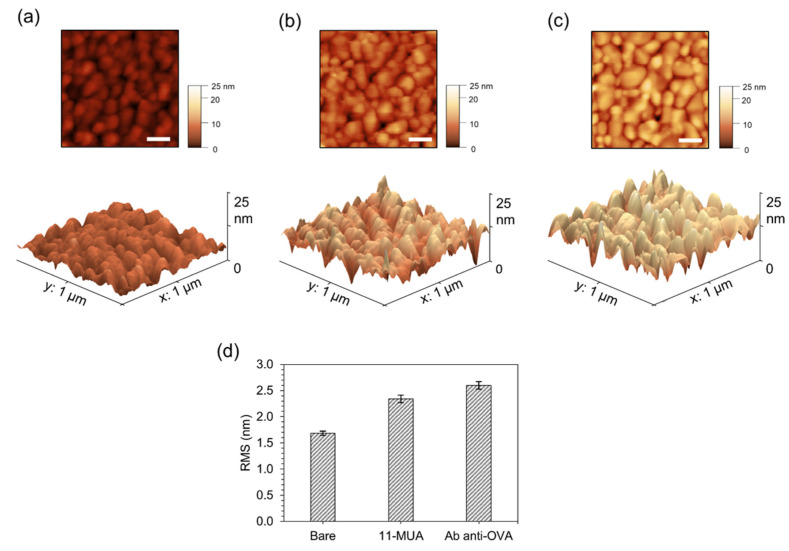
AFM analysis of pAb anti-OVA immobilisation on the electrochemical immunosensor. The 2D and 3D AFM morphology scans (1 μm × 1 μm) acquired in tapping mode of (**a**) the bare gold electrode, 11-MUA-modified gold surface (**b**) before and (**c**) after pAb anti-OVA incubation. The white scale bar in the 2D image corresponds to 20 nm. (**d**) Bar plot of the root-mean-squared roughness (RMS) of each analysed surface and presented as the average ± the SD (*n* = 10).

Since the electrochemical response depends on the morphological characteristics of the surface electrode, AFM is useful to evaluate the immobilisation of the pAbs anti-OVA on the impedimetric immunosensor. In this respect, the root mean square (RMS) value of the height irregularities was chosen to characterise the immunoassay surface roughness [31]. The following AFM analysis was carried out on the same chip at different stages of the pAb anti-OVA immobilisation process to avoid the fluctuations of the surface roughness due to the inter-batch variability of the electrochemical surface sensors. Figure 4a illustrates the AFM morphology map of 1 μm × 1 μm in size acquired on the working area of the bare gold-based electrode. The surface of the electrochemical sensor was characterised by a low roughness (RMS = 1.7 ± 0.1 nm) and showed the typical granular structure, consisting of nearly spherical nanoparticles, which was strictly connected to the gold deposition process parameters used by Micrux manufactory [32]. The sensor was, then, functionalised with an 11-MUA SAM, as described in Section 2.4. The evidence that the gold surface was chemically modified was emphasised by the significant change of the surface roughness (RMS = 2.3 ± 0.1 nm) as observed in the AFM morphology of the surface (Figure 4b). Finally, in Figure 4c is shown the AFM morphology map of the 11-MUA-modified gold surface after the incubation of the pAbs anti-OVA. As highlighted in the bar plot of Figure 4d, the deposition of the consecutive layers on the electrode affected the RMS value of the surfaces. In particular, the increment of the RMS value of the pAb anti-OVA surface (2.6 ± 0.1 nm) compared to the 11-MUA-modified gold surface and the bare electrode surface was comparable to the roughness change detected in similar studies [33,34]. The AFM results further assessed the complete immobilisation process of the pAbs anti-OVA as also confirmed by the impedimetric experiments.

### 3.4. Electrochemical Characterisation of the Immunosensor Performance

**Figure 5:**
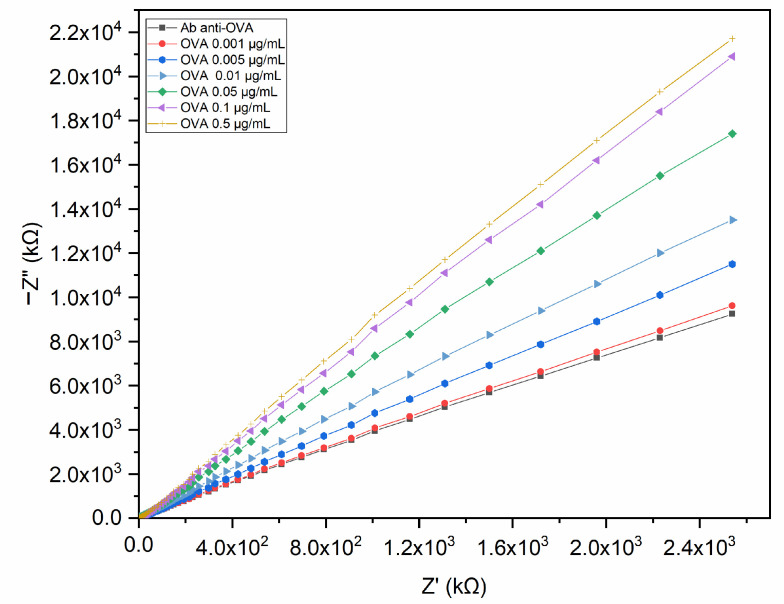
Nyquist plot of the pAb-anti-OVA-based immunosensor tested on OVA in PBS. The impedance of the electrochemical system increased at increasing concentrations of OVA.

The electrochemical performance of the immunosensor was examined by non-Faradaic EIS for detecting OVA dissolved in PBS. Unlike the Faradaic approach, a non-Faradaic response shows high impedance values given that no redox species assist the charge transfer between the interfacial layers [35]. The impedance response was recorded starting from the lowest (0.001 μg/mL) to the highest (0.5 μg/mL) concentration of OVA. The insulating effect of the sensing surface and the OVA binding on the modified electrode generated variations in the double-layer capacitance (C_dl_). The binding phenomena occurring at the electrode interface can be observed in the Nyquist plot as an increase in the imaginary part of impedance at increasing OVA concentrations (Figure 5).

**Figure 6:**
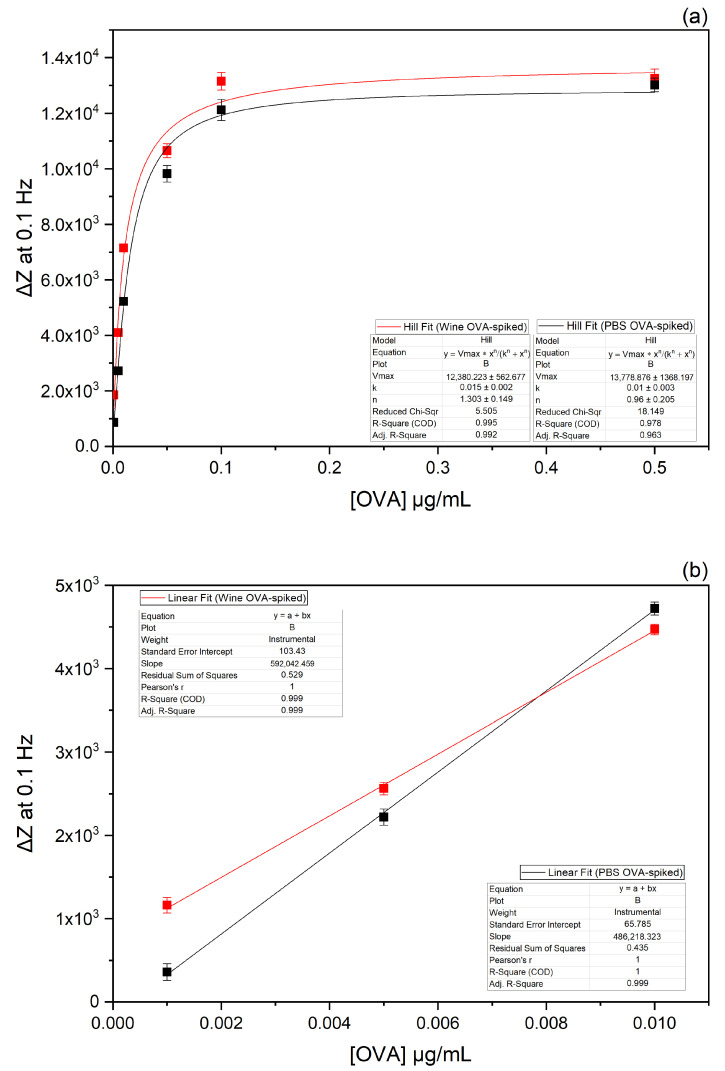
Plots of the change in impedance (ΔZ) at 0.1 Hz versus OVA concentration dissolved in PBS (in black colour) and OVA concentration in spiked white wine (in red colour). The binding curves were obtained through a non-linear fitting function (**a**). The calibration curves of the immunosensor were obtained through a linear fitting function (**b**).

As the maximum impedance variation was observed at 0.1 Hz, the impedance values registered at this frequency allowed us to obtain the binding curve described by a non-linear fitting function. Figure 6a shows the plot of the change in impedance at 0.1 Hz expressed as ΔZ (Z_OVA_ − Z_blank_) versus OVA concentration.

A linear correlation was observed in the range of 0.001 to 0.01 μg/mL, while for higher concentrations (from 0.05 to 0.5 μg/mL), no linear trend was noticeable, due to the saturation of the binding sites. Therefore, the calibration curve was calculated in the linear range to determine the detection limit (LoD) of the immunosensor in PBS (Figure 6b). Each point represents the average of three replicates, and the error bars are the standard deviations of the mean (SDs ≤ 380 ΔZ). The LoD, calculated by 3.3 S/b, where S is the standard deviation of the y-intercept of the linear regression and b is the slope of the linear range [27], was estimated to be 0.0008 µg/mL, with a response time of 15 min, including the incubation time. As a control, the surface was tested at increasing concentrations of BSA, and no significant impedance variations were registered (Figure S2).

### 3.5. Electrochemical Characterisation of the Immunosensor Performance on Real Matrix

**Figure 7:**
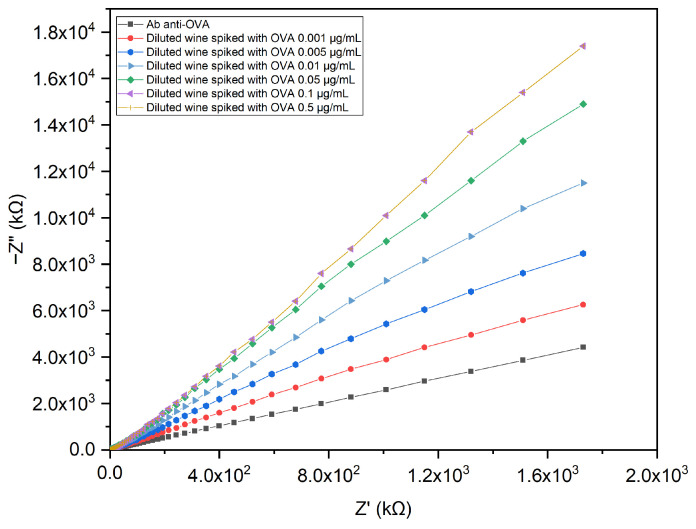
Nyquist plot of the pAb-anti-OVA-based immunosensor tested with white wine diluted in PBS and spiked with OVA.

In order to explore the application of the immunosensor on a real matrix, impedance measurements were conducted on white wine diluted in PBS and spiked with different concentrations of OVA (0.001, 0.005, 0.01, 0.05, 0.1, and 0.5 μg/mL) as described in Section 2.7. The impedimetric response of the electrode reported in Figure 7 displays that the immunosensor was able to detect OVA even in wine.

Taking into consideration that the maximum impedance variation was at 0.1 Hz, the impedance values registered at this frequency were used to obtain the binding curve by applying a non-linear fitting function. The plot of the change in impedance at 0.1 Hz expressed as ΔZ (Z_OVA in wine_ − Z_blank_) versus the OVA concentration is shown in Figure 6a.

The calibration curve (Figure 6b) was calculated in the linear range of 0.001 to 0.01 μg/mL to determine the detection limit of the immunosensor in wine. The graph shows standard deviation error bars from the mean of triplicate measurements (SDs ≤ 340 ΔZ). The LoD, calculated by 3.3 S/b [28], was estimated to be 0.001 µg/mL, with a response time of 15 min, including the incubation time. By considering the 200-fold dilution used in the measurements, the LoD of the assay in white wine was 0.20 µg/mL, a value that is compliant with the detection limit recommended by the OIV (i.e., 0.25 μg/mL) for the quantification of allergens in wine [7].

Reproducibility data calculated in terms of the relative standard deviation (RSD) are reported in Table S1. The immunosensor, stored in PBS buffer at 4 °C, showed stability for 2 weeks after the functionalisation procedure.

Table 1 depicts a comparison of the analytical performance of the immunosensor with other biosensors for OVA detection previously reported. The detection limit obtained in this work is compatible with the limit established by the resolution OIV/COMEX 502–2012 for quantifying allergens in wine. In addition, the immunosensor presents advantages such as a rapid response, the ease of the functionalisation process, the need for micro volumes of the sample, an assay time of 15 min, and affordability.

## 4. Conclusions

In this work, we presented a non-Faradaic impedimetric immunosensor to monitor the presence of OVA in white wine. The primary purpose of this study was to explore the feasibility of using an impedimetric pAb-anti-OVA-based biosensor on a real matrix without the use of external probes and the need for sample pre-treatment. The immunosensor showed a sensitivity of 0.20 µg/mL, which is compatible with the limit established by the resolution OIV/COMEX 502–2012 for quantifying the presence of allergens in wine. Although the developed biosensor presents some advantages with respect to the biosensors for OVA detection present in the literature (such as affordability, a good response time, and the direct monitoring of OVA in diluted samples), additional efforts will be devoted to improving the performance of the biosensor.

## Figures and Tables

**Table 1:** Performance comparison of different biosensors for OVA detection.

Sensing Substrate	Technique	Linear Range	LoD	Assay Time (min)	Number of Steps	References
GO/screen-printed carbon	Differential pulse voltammetry	1 pg/mL–0.5 μg/mL	0.83 pg/mL	60	5	Eissa et al. (2013) [15]
Dextran-coated sensor chips (CM5)	Surface plasmon resonance (SPR)	0.03–0.2 μg/mL	0.6 μg/mL	–	4	Pilolli et al. (2015) [16]
Screen-printed platinum	Linear sweep voltammetry	0.5–9.5 μg/mL	0.2 μg/mL	60	4	Čadková et al. (2015) [17]
Graphene/screen-printed carbon electrodes	Amperometry	0.01–10 pg/mL	0.2 fg/mL	60	6	Baldo et al. (2021) [18]
Thin-film gold single electrodes	Non-Faradaic impedance spectroscopy	0.001–0.01 μg/mL	0.2 µg/mL	15	4	This work

## Data Availability

The data presented in this study are available within the article and its Supplementary Materials. Other data that support the findings of this study are available upon request from the corresponding author.

## Supplementary Materials

The following Supporting Information can be downloaded at: https://www.mdpi.com/article/10.3390/bios13070669/s1, Figure S1: EIS spectra acquired at different AC potentials in the frequency range of 0.1 to 100,000 Hz.; Figure S2: Immunosensor BSA cross-reactivity.; Table S1: Immunosensor reproducibility data.
