# Supplementary material for: An Impedance-Based Immunosensor for the Detection of Ovalbumin in White Wine
Source: Biosensors (Basel). 2023 Jun 22;13(7):669. doi: 10.3390/bios13070669 (PMC10377481; doi:10.3390/bios13070669)
Supplement: Supplementary file 1 [file biosensors-13-00669-s001.zip › biosensors-2431949-supplementary.pdf]

biosensors

Supporting Information

An impedance-based immunosensor for the detection of ovalbumin in white wine

Alessia Calabrese [1], Alessandro Capo [1], Angela Capaccio [1], Elettra Agovino [1], Antonio Varriale [1], Michelangelo Pascale [2], Sabato D'Auria [3,*] and Maria Staiano [2]

[1] Institute of Food Science, URT-CNR, 80126 Naples, Italy; alessia.calabrese@isa.cnr.it(A.C.); alessandro.capo@isa.cnr.it (A.C.); angela.capaccio@isa.cnr.it (A.C.); e.agovino95@libero.it (E.A.); antonio.varriale@isa.cnr.it (A.V.)
[2] Institute of Food Science, CNR, 83100 Avellino, Italy; michelangelo.pascale@cnr.it (M.P.); maria.staiano@isa.cnr.it (M.S.)
[3] Department of Biology, Agriculture and Food Science, CNR, Piazzale Aldo Moro, 7, 00185 Rome, Italy
* Correspondence: sabato.dauria@cnr.it; Tel.: +39-3683422770

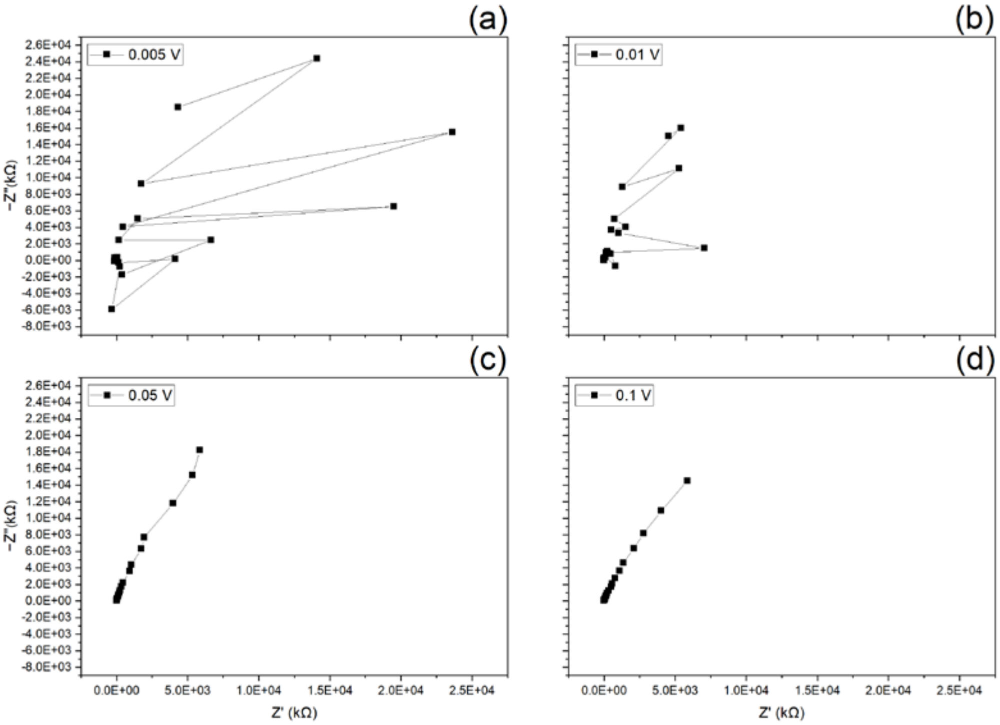

Figure S1. EIS spectra acquired at different AC potentials in the frequency range of 0.1 to 100,000 Hz. The graph shows the impedance response of the immunosensor at 0.005 V **(a)**, 0.01 V **(b)**, 0.05 V **(c)**, and 0.1 V **(d)**. Linearity and the best signal-to-noise ratio were obtained at t 0.1 V.

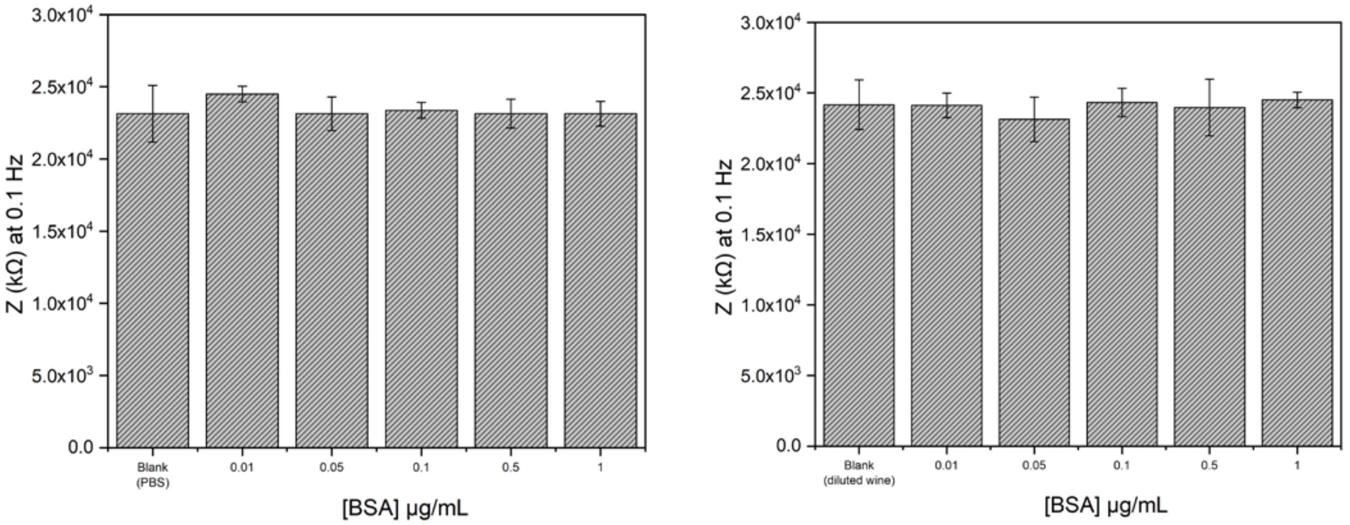

Figure S2. Immunosensor BSA cross-reactivity. The figure shows the impedance response (Z) of the immunosensor exposed to increasing concentrations of BSA dissolved in PBS (**a**) and in diluted white wine (**b**).

Table S1. Immunosensor reproducibility data. The Relative Standard Deviation of each OVA measurement was calculated on six different immunosensors. The RSD data were pooled in two groups to highlight the reproducibility of the immunosensor when exposed to OVA dissolved in PBS and to diluted wine spiked with OVA.

[OVA] μg/mL	RSD (PBS OVA-spiked)	RSD (wine OVA-spiked)
0 [1]	7.3 %	8.4 % [2]
0.001	27.7 %	8.1 %
0.005	4.4 %	2.9 %
0.01	1.6 %	1.4 %
0.05	3.2 %	2.3 %
0.1	3.3 %	2.4 %
0.5	1.9 %	2.6 %

[1] Blank
[2] The blank measurement in the presence of PBS was performed after a 10 minutes-incubation with diluted white wine (not spiked) and extensive washing with PBS.

Disclaimer/Publisher's Note: The statements, opinions and data contained in all publications are solely those of the individual author(s) and contributor(s) and not of MDPI and/or the editor(s). MDPI and/or the editor(s) disclaim responsibility for any injury to people or property resulting from any ideas, methods, instructions or products referred to in the content.
